# Using advanced neuroimaging and bioinformatics methods to study brain-behaviour relationships

**DOI:** 10.1192/j.eurpsy.2025.67

**Published:** 2025-08-26

**Authors:** S. Frangou

**Affiliations:** 1 Psychiatry, Icahn School of Medicine at Mount Sinai, New York, United States; 2 Psychiatry, University of British Columbia, Vancouver, Canada

## Abstract

**Abstract:**

Understanding the complex relationships between brain structure, function, and behavior is a central challenge in neuroscience. This presentation aims to showcase the transformative potential of neuroimaging and bioinformatics in bridging the gap between neural mechanisms and behavior, ultimately advancing our understanding of the human brain and informing precision medicine. Recent advancements in neuroimaging and bioinformatics enable researchers to explore these relationships with unprecedented precision and scale. This presentation will provide an overview of how neuroimaging modalities can be integrated with advanced bioinformatics tools, including machine learning to uncover novel brain-behavior associations. We will discuss key applications of these methods for neuropsychiatric disorders and specific examples will be used to highlight how combining neuroimaging data with bioinformatics pipelines enhances our ability to measure brain organization at the level of a single individual. Additionally, challenges such as data complexity, standardization, and interpretability will be addressed, alongside strategies to overcome them.

**Disclosure of Interest:**

None Declared

